# Occurrence and severity of non-occlusive mesenteric ischemia (NOMI) after cardiovascular surgery correlate with preoperatively assessed FGF-23 levels

**DOI:** 10.1371/journal.pone.0182670

**Published:** 2017-08-08

**Authors:** Jonas Stroeder, Matthias Klingele, Hagen Bomberg, Stefan Wagenpfeil, Arno Buecker, Hans-Joachim Schaefers, Marcus Katoh, Peter Minko

**Affiliations:** 1 Department of Diagnostic and Interventional Radiology, Saarland University Medical Center, Homburg, Germany; 2 Department of Internal Medicine IV - Nephrology and Hypertension, Saarland University Medical Center, Homburg, Germany; 3 Department of Anaesthesiology, Critical Care and Pain Medicine, Saarland University Medical Center, Homburg, Germany; 4 Institute for Medical Biometry, Epidemiology and Medical Informatics, Saarland University, Saarland University Medical Center, Homburg, Germany; 5 Department of Thoracic and Cardiovascular Surgery, Saarland University Medical Center, Homburg, Germany; 6 Helios Klinikum Krefeld, Clinic for Diagnostic and Interventional Radiology, Krefeld, Germany; Hospital Universitario de la Princesa, SPAIN

## Abstract

**Purpose:**

To evaluate the value of preoperatively assessed fibroblast growth factor 23 (FGF-23) levels and to correlate FGF-23 with angiographic findings in non-occlusive mesenteric (NOMI) ischemia using a standardized scoring system.

**Materials and methods:**

Between 2/2011 and 3/2012 a total of 865 patients (median age: 67 years) underwent cardiovascular surgery during this ethics committee approved, prospective study. 65 of these patients had clinical suspicion of NOMI and consequently underwent catheter angiography of the superior mesenteric artery. Images were assessed using a standardized reporting system (Homburg-NOMI-Score). These data were correlated to following preoperative parameters of kidney function: cystatin C, creatinine, FGF-23 and estimated glomerular filtration rate (eGFR), and outcome data (death, acute renal failure) using linear and logistic regressions, as well as nonparametric tests.

**Results:**

Significant correlations were found between FGF-23 and the angiographic appearance of NOMI (p = 0.03). Linear regression analysis showed no significant correlation to the severity of NOMI with creatinine (p = 0.273), cystatin C (p = 0.484), cystatin C eGFR (p = 0.914) and creatinine eGFR (p = 0.380). Logistic regression revealed a significant correlation between death and the Homburg-NOMI-Score (p<0.001), but not between development of NOMI and acute renal failure (p = 0.122). The ROC Analysis yielded an area under the curve of 0.695 (95% CI: 0.627–0.763) with a sensitivity of 0.672 and specificity of 0.658.

**Conclusions:**

FGF-23 significantly correlates with the severity of NOMI, which is in contrast to other renal function parameters. The applied scoring system allows to predict mortality in NOMI patients.

## Introduction

Non occlusive mesenteric ischemia (NOMI) is a rare (incidence around 1%)[1] but feared (mortality up to 90%)[2] disease, in which the perfusion of the intestine is limited. It is believed, that a reduction of splanchnic blood flow causes an intestinal ischemia. This can lead to severe damage in the mucosal layer resulting in bacterial translocation and bacteremia, which finally may lead to multi organ failure[3]. In the diagnosis of NOMI, invasive angiography with digital subtraction angiography (DSA) is considered to be the gold standard[4]. The therapy for NOMI is to correct underlying vascular pathology and diffuse vasospasm with application of vasodilatators[5–7], which is done selectively through an angiographically placed catheter in the superior mesenteric artery (SMA). Clinical presentation of NOMI is manifold and poses a diagnostic challenge, as clinical signs such as oliguria, rising serum lactate levels, decreased blood oxygenation, hypertension and abdominal pain are nonspecific. Moreover in patients who receive an analgosedation therapy, abdominal pain might be completely masked. Several risk factors for NOMI have been identified in the past including renal insufficiency[3,8,9] and coronary heart disease[1,9]. These risk factors also seem to influence the blood level of fibroblast growth factor 23 (FGF-23)[10].

FGF-23 is a protein consisting of 251 amino acids[11], which is mainly synthesized in the osteocytes and osteoblasts in the bone[12]. FGF-23 is an endocrine signaling protein[13], which plays an important role in several metabolic pathways, including the Vitamin D and phosphate regulation. Increased blood levels of FGF-23 are described in patients with chronic renal disease and to be a predictor of the mortality in dialysis patients [14]. This positive association of FGF-23 levels and mortality are in contrast to other cardiovascular markers, which seem to be protective of cardiovascular death in this group of patients[14]. Furthermore, some studies even show that elevated FGF-23 levels were associated with an increased risk for cardiovascular death, regardless of chronic kidney disease[10]. Additionally, it has recently been shown that FGF-23 is a predictor of death in patients suffering from myocardial infarction and cardiogenic shock[15].

The aim of our study was to prospectively evaluate the value of preoperatively assessed FGF-23 levels for NOMI and to correlate FGF-23 levels with angiographic findings using a standardized scoring system. Furthermore we investigated the clinical value of angiography in predicting the outcome in patients after cardiovascular surgery with clinical suspicion of NOMI.

## Materials and methods

### Selection of patients

Between February 2011 and March 2012 all patients undergoing cardiac surgery at our department of Thoracic and Cardiovascular Surgery were screened for participation in a prospective observational study. The study was approved by the local ethics committee. Finally, 865 patients could be included and underwent preoperative measure of blood levels of FGF-23, creatinine and cystatin C. Written informed consent was obtained from all participants. Exclusion criteria were age < 18 years and cases in which informed consent to participate in the study could not be obtained. The recruited patient collective is the same as used by Speer et al.[16] who focused on cardiovascular mortality, but as we focused on the severity of NOMI we based our research on the angiographic findings. Consequently, we had to exclude several patients for different reasons. For the actual evaluation, 4 patients had to be excluded due to missing laboratory data, an additional 22 patients had to be excluded because of technical reasons leading to incomplete angiographic data. Of these 839 patients 65 patients (24 female; 41 male) were under clinical suspicion of NOMI. They suffered from at least one of the following: new onset of oliguria (urine output <0.5 ml/kg/h for at least 6 h) or anuria, distention of the abdomen with decreased or absent bowel sounds, elevated serum lactate levels > 5.0 mmol/l or metabolic acidosis (base excess > -5 mmol/l). These patients will be called the “NOMI-Group” and underwent a diagnostic angiogram of the superior mesenteric artery as soon as the clinician suspected NOMI. The other patients are consequently considered the “Non-NOMI-Group”. Patient characteristics are displayed in Table 1.

**Table 1:** Patient characteristics.

	All Patients									p-Value (2-sided)
	All			No NOMI			NOMI			
	n	Median	Range	n	Median	Range	n	Median	Range	
Age	839	67.0	[19.3;88.5]	774	66.1	[19.3; 88.5]	65	74.4	[46.8; 88.2]	<0.001*
BMI	839	27.2	[16.5;46.4]	774	27.1	[16.5; 45.7]	65	28.7	[19.4; 46.4]	0.133
FGF-23 [RU/ml]	839	64.2	[7.3;53110]	774	61.9	[7.3; 53110]	65	108.5	[22.5; 16959.5]	<0.001*
Kreatinin [mg/dl]	839	1.0	[0.5;11.4]	774	1.0	[0.5; 11.4]	65	1.2	[0.7; 3.8]	0.001*
Cystatin C [mg/l]	838	1.06	[0.4;7]	773	1.04	[0.4; 7]	65	1.52	[0.4; 5.4]	<0.001*
Urea [mg/dl]	839	40.0	[13;222]	774	39.0	[13; 222]	65	49.0	[18; 192]	<0.001*
GFR MDRD	839	72.95	[3.63; 146.77]	774	73.83	[3.63; 146.77]	65	56.82	[17.27; 117.04]	<0.001*
CKD-EPI cystatin C	838	68.05	[5.49; 156.64]	773	70.31	[5.49; 156.64]	65	39.48	[8.31; 144.81]	<0.001*
CKD-EPI creatinin	839	72.02	[3.21; 131.72]	774	73.15	[3.21; 131.72]	65	52.53	[16.04; 101.49]	<0.001*
Hospital stay [days]	839	9	[1;84]	774	9	[1; 84]	65	16	[2; 77]	<0.001*
ICU stay [days]	839	1	[0;54]	774	1	[0; 54]	65	7	[0; 46]	<0.001*
OP-duration [min]	839	161	[45;651]	774	160	[45; 651]	65	206	[77; 450]	<0.001*
LOS	839	13	[0;132]	774	13	[0; 112]	65	24	[0; 132]	<0.001*

Patient characteristics and results of Mann-Whitney-U-Test performed to compare Groups with and without NOMI. Significant differences between groups are marked with an asterisk (*) in the last column. n indicates absolute frequency.

### Assessment of laboratory values

The blood samples were each taken under standard conditions alongside other routine preoperative laboratory assessments directly prior to surgery.

The blood samples for the FGF-23 measurement were centrifuged at 2800 x g for ten minutes at 4°C. Supernatants were stored in aliquots at −80°C. C-terminal FGF-23 levels were measured from plasma samples by ELISA (Immutopics, San Clemente, CA, USA) in relative Units per milliliter (RU/mL).

Serum creatinine was measured using a Jaffe-Test. The assessed creatinine values were used to calculate the Creatinine-GFR values using the CKD-EPI formula[17]. Cystatin C was measured using a particle-enhanced turbodimetric test and the Cystatin C-GFR was consequently calculated using the CKD-EPI formula for Cystatin C.

### Assessment of NOMI

**Fig 1:**
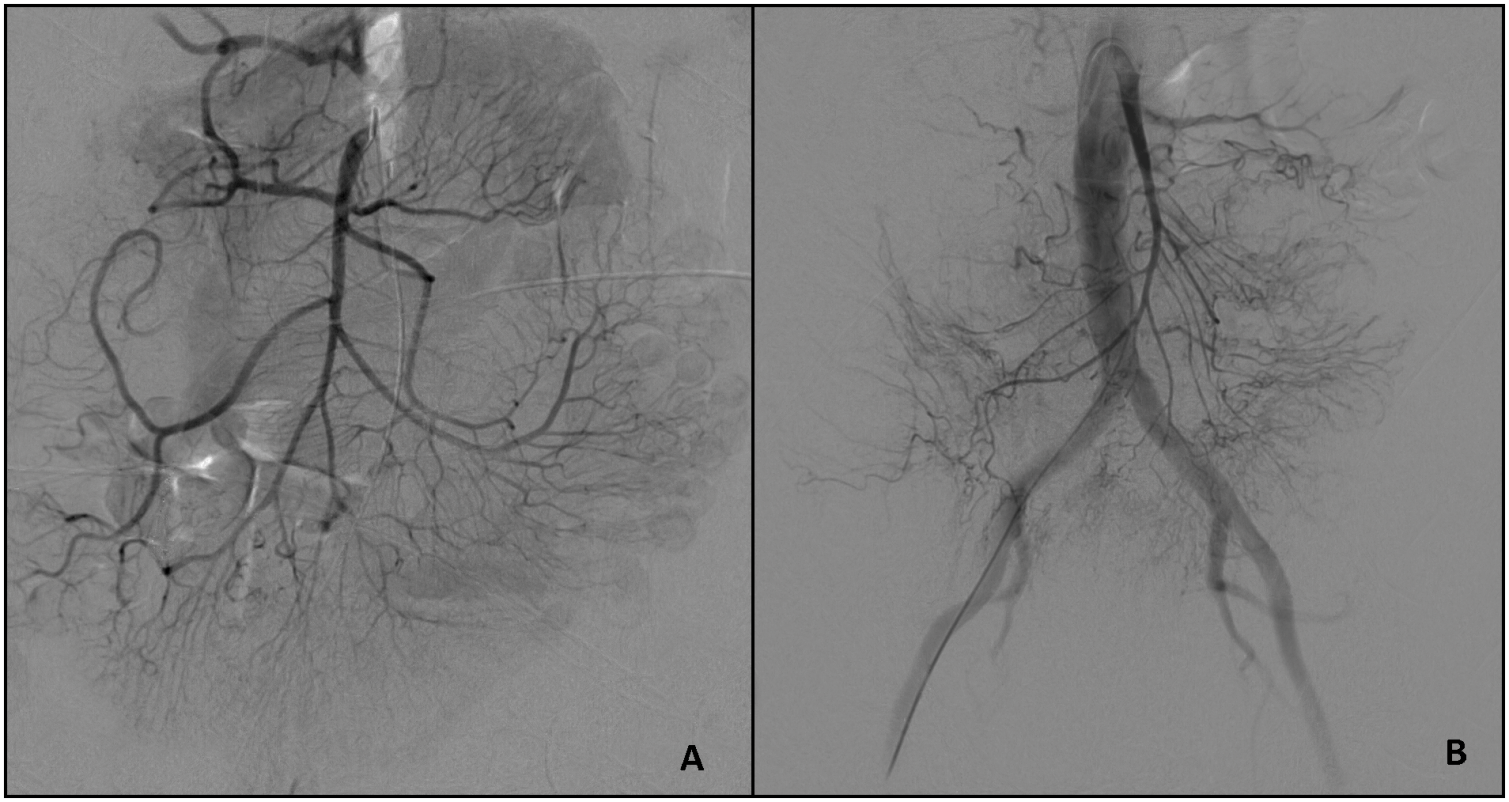
Angiographic findings in mild and severe NOMI. Comparison of angiographic findings in a patient with a low FGF-23 value of 29 (**A**, Score of 1 due to a slight reflux into the aorta and no signs of vasospasm and no delayed portal vein filling) and a patient with a high FGF-23 value of 4636 and a severe NOMI (**B**, Score of 9 due to severe vasospasm and reflux into the aorta as well as delayed portal vein filling).

All of these 65 patients underwent a routine standardized invasive angiographic procedure through a transfemoral 5F sheath placed in the common femoral artery. Subsequently the SMA was catheterized using a 4F catheter (Cordis, Bridgewater, USA) with a cobra tip configuration. The tip of the catheter was placed in the main stem of the SMA. A DSA was performed after application of 25 ml Imeron 300 (Bracco, Italy) at a rate of 5 ml/s in every case (Fig 1). The injection was performed using a single-head injector Injektron 82 (Medtronic, Saarbruecken, Germany). The series was recorded using a framerate of 2 frames per second until the portal vein was contrasted. Afterwards, these angiograms were assessed and consecutively scored by two experienced radiologists on consensus basis, using a modification of the „Homburg-NOMI-Score”published in 2012[18], the simplified version of the score published in 2014[19]. This simplified NOMI score (consisting of three categories namely vessel morphology, reflux of contrast medium into the aorta and time to portal vein filling) was used because it showed higher interobserver correlations and is easier to apply than the original score[18]. Furthermore, previously conducted studies showed even better correlation to mortality than the complete NOMI score[19]. Therefore the term Homburg-NOMI-Score will be used for the applied simplified version of the NOMI scoring system.

### Statistics

Comparison between the NOMI and the Non-NOMI group was performed using the Mann-Whitney-U-Test and Chi-square-Test. In addition, a ROC- Analysis for the FGF-23 values was conducted. NOMI-Score values were then investigated with respect post-operative mortality rate using logistic regression analysis. Cox regression was used to analyze time-to-event data. In addition, the influence of preoperative blood values (serum levels of FGF-23, cystatin C, creatinine as well as calculated GFR for serum creatinine and for serum cystatin) on the angiographic score was investigated using linear regression analysis. All analyses were performed using SPSS Version 20 (IBM, Armonk, NY, USA). We used the natural logarithm (ln) of the FGF-23 values as they provided a better fit of the linear regression models. Consequently, this enabled the use of these models to assess the FGF-23 values in combination with the values resulting from the NOMI score. Any p-values given are two-sided and subject to a significance level of 0.05.

## Results

The patients under suspicion of NOMI had an average Homburg-NOMI-Score of 3.2 (range 1–7, median 3).

No significant difference between the patients suffering from NOMI and those without NOMI was found concerning sex distribution (p = 0.302) and BMI (median: 28.7 vs. 27.1; p = 0.133).

There was a significant difference between the two groups concerning age (median: 66.1 vs. 74.4; p<0.001), FGF-23 (median: 61.9 vs. 108.5; p<0.001), Creatinine (median: 1.0 vs. 1.2; p<0.001), Cystatin C (median: 1.04 vs. 1.52; p<0.001), Creatinine-GFR (median: 73.15 vs. 52.53; p<0.001) and Cystatin C-GFR (median: 70.31 vs. 39.48; p<0.001). All these values were significantly higher in the NOMI group, except for the calculated GFRs which were lower. For further details see Table 1.

The ROC Analysis concerning FGF-23 values and the occurrence of NOMI yielded an area under the curve of 0.695 (95% CI: 0.627–0.763) with a sensitivity of 0.672 and specificity of 0.658 for a FGF-23 value of 82.545.

In the NOMI-group, significant correlations were found between FGF-23 and the Homburg-NOMI-Score (p = 0.03). No significant correlation to the severity of NOMI was found for creatinine (p = 0.273), cystatin C (p = 0.484), cystatin C eGFR (p = 0.914) and creatinine eGFR (p = 0.380) as shown in Table 2. Patients without clinical suspicion of NOMI did not undergo angiography and therefore could not be evaluated in this regard.

**Table 2:** Results of linear regression analysis.

	NOMI Score		Vessel Morphology		CM-Reflux to Aorta		Time to portal vein filling	
	p-Value	Estimated Effect	p-Value	Estimated Effect	p-Value	Estimated Effect	p-Value	Estimated Effect
Ln FGF-23	0.030*	0.296	0.382	0.073	0.065	0.128	0.086	0.096
Cystatin C [mg/l]	0.484	0.201	0.872	0.028	0.981	-0.003	0.127	0.177
Kreatinin [mg/dl]	0.273	0.478	0.143	0.383	0.290	-0.233	0.061	0.328
CKD-EPI cystatin C	0.914	-0.001	0.960	0.000	0.941	0.000	0.776	-0.001
CKD-EPI creatinin	0.380	-0.009	0.204	-0.008	0.381	0.005	0.166	-0.006

NOMI-Score correlates significantly with FGF-23, but not with other renal function parameters. Significant findings are marked with an asterisk (*).

Correlation of FGF-23 and time to portal vein filling showed a tendency with a p-value of 0.09, but did not reach statistical significance. The same is true of the reflux of contrast medium into the aorta (p = 0.07).

Logistic and Cox regression models revealed a significant correlation between death and the Homburg-NOMI-Score (p<0.001). No significant correlation was found for the development of acute renal failure (p = 0.144) using logistic regression analysis, but a correlation was found using Cox regression analysis (p = 0.037) as can be seen in Table 3.

**Table 3:** Results of logistic and Cox regression analysis.

Logistic Regression			Cox Regression	
	NOMI Score		NOMI Score	
	P-value	Odds Ratio	P-value	Hazard Ratio
ARF	0.144	1.391	0.037*	1.193
Death	< 0.001*	2.957	< 0.001*	2.425

NOMI-Score correlates significantly with death, but not with acute renal failure (ARF). Significant findings are marked with an asterisk (*).

## Discussion

NOMI is a severe condition, which might occur after cardiovascular surgery or in the course of dialysis therapy, but hardly any predictors are known thus far. Various studies about risk factors for NOMI have been conducted and several risk factors have been successfully identified[1,20]. Through the course of this study, significant differences between the NOMI and Non-NOMI-Group regarding age, cystatin, creatinine and duration of the operation were found. These significant differences between the groups among risk factors for NOMI are similar to the risk factors identified by Groesdonk et al. in 2013[1]. Among these risk factors several parameters for cardiac and renal insufficiency have been reported. As FGF-23 levels are known to be elevated in these conditions, we suspected them to be elevated in patients at risk for NOMI as well. We could show that preoperative FGF-23 values of patients with angiographic prove of NOMI are significantly higher than those of Patients showing no clinical signs of NOMI. Additionally, we were able to show for the first time that the preoperative FGF-23 levels correlated with the severity of the NOMI seen by DSA. These findings lead to the conclusion that the preoperative determination of FGF-23 might be of value in the prediction of the occurrence of postoperative NOMI, even though only a sensitivity of 67% and a specificity of 66% could be identified. Whether a combination of FGF-23 and other parameters or a postoperative FGF-23 leads to a better sensitivity and specificity has still to be investigated.

The assessment of the severity of NOMI using the Homburg-NOMI-Score has shown a highly significant (p<0.001) correlation to the event of death during the postoperative period [19]. These data could be confirmed in our patient collective and thus further support the quality and value of the applied scoring system. Reproducibility of the score has previously been reported which is needed to have a clinical impact. In addition, a standardized reporting system will help clinicians to act accordingly and thus facilitate standardization of clinical pathways in this multifactorial disease. The reproducibility enables the clinician to be able to use follow-up examinations to see effects and adapt the applied therapy, which is currently under investigation.

In current literature there is a certain interest to perform a computed tomography as a first line technique in such a setting [21], but it has not found entry into current guidelines and it does not allow for immediate treatment, if NOMI is detected.

As the pathophysiology of NOMI is not completely understood, it is known to be a multifactorial disease in which organ systems interacting with FGF-23 might play a major role. However, FGF-23 might be just a marker and not the cause of NOMI. Furthermore, the preoperative levels of FGF-23 might as well indicate a preoperative condition of an organ malfunction that severely aggravates during/after cardiovascular surgery and the following ICU stay. Assessing the preoperative values of FGF-23 was used in this study to identify a parameter mirroring the cardiac and renal health condition of the patients. Other renal function parameters were also taken into account, to avoid assessing the severity of their impairment due to the surgical procedure. By correlating the preoperative blood levels of FGF-23 to the severity of NOMI we have found a parameter which could be useful in clinical practice. It might be advantageous to preoperatively know, which patients have an increased risk to suffer from severe NOMI. These patients could profit from added caution to detect and treat the NOMI as early as possible. This also poses one limitation of the study, as no FGF-23 values were obtained directly before the angiography and thus no acute change in FGF-23 levels were investigated. Furthermore it could have been of interest to also evaluate postoperative FGF-values but as we were looking for a predictor, this can be done in following studies.

NOMI has been shown to be in great parts related to cardiovascular factors, especially those resulting in low cardiac output, but has also been shown to correlate with renal insufficiency[11,15,17]. Other results supporting these findings show FGF-23 to be increased in patients with renal insufficiency and at elevated risk of NOMI which contributes to overall mortality; a correlation of FGF-23 and cardiovascular mortality had already been shown by Speer et al[16]. A direct correlation between death and the FGF-23 levels could not be shown in this study, but a tendency could be detected to support this theory as the calculated p-value was 0.07. This could be due to the nature of the FGF-23 blood levels to be influenced by the working state of more than one organ system. Further pathophysiological research taking into account the blood levels of FGF-23 should be conducted.

There are no data available about correlations between preoperative FGF-23 and the mortality of patients suffering from an untreated NOMI. These data will be difficult to obtain, as therapeutic approaches have been identified to improve patient conditions. Consequently, it would be unethical not to treat patients with NOMI accordingly. There was no angiography performed in the non-NOMI-group because these patients should not be put in jeopardy to suffer a complication of angiographies, even though these are rare. Consequently, some of these patients might have suffered from a subclinical course of a NOMI.

In conclusion, this prospective study showed that preoperative FGF-23 significantly correlates with the angiographic signs for severity of NOMI and the applied Homburg-NOMI-Score allows to predict fatal outcome in NOMI patients thus FGF-23 proved to be an important preoperative marker in patients undergoing cardiovascular surgery and might help to identify patients with a high risk of NOMI and to improve postoperative outcome; catheter angiography should be analyzed using the previously published score.
